# Case report: aortic root thrombus presenting with syncope and embolic events

**DOI:** 10.1093/ehjcr/ytag715

**Published:** 2026-09-19

**Authors:** Ai Phi Thuy Ho, Eirik Brekka Tjønnfjord, Ole-Christian Walter Rutherford

**Affiliations:** Department of Cardiology, Kalnes Hospital, 1714 Grålum, Norway; Emergency Department, Kalnes Hospital, 1714 Grålum, Norway; Department of Cardiology, Kalnes Hospital, 1714 Grålum, Norway

**Keywords:** Aortic root thrombus, Systemic embolization, Non-infective thrombus, Case report, Cardiac tumours

## Abstract

**Background:**

Aortic root thrombus is a rare but potentially life-threatening condition, often associated with systemic embolization or obstruction of coronary arteries (myocardial infarction). The most common reason is a complication of left ventricular assist device (LVAD) implantation, but it may occur in the absence of LVAD, for instance in structural valve disease or prothrombotic disorders (antiphospholipid syndrome, haemolytic diseases, and myeloproliferative disease, making diagnosis challenging.

**Case summary:**

We report the case of a 47-year-old man with a history of deep venous thrombosis who presented with syncope, anaemia, and new-onset atrial fibrillation. Initial evaluation revealed elevated troponin, cerebral and splenic embolic lesions, and a mobile aortic root mass measuring 2.3 × 1.3 cm. Despite extensive work-up, no autoimmune or haematological disorder was identified. Surgical removal of the thrombus was performed. His postoperative course was complicated by pericardial tamponade requiring drainage but was otherwise favourable. He was discharged on long-term anticoagulation with preserved cardiac function.

**Discussion:**

Aortic root thrombus may present with systemic embolization in otherwise healthy individuals without identifiable thrombophilia. Early recognition and surgical intervention are essential when thrombus size, mobility, and embolic risk are high.

Learning points

- Aortic root thrombus can present with systemic embolization even in patients without identifiable thrombophilia, making diagnosis challenging.
- Early recognition with multimodal imaging and timely surgical intervention is crucial to prevent life-threatening complications and optimize outcomes.

## Introduction

A mass in the aortic root may represent thrombus, tumours, infectious vegetations, or iatrogenic artefacts.^1,2^ Aortic root thrombi are rare but may occur even in the absence of significant atherosclerotic disease.^3,4^ Accurate diagnosis is essential to guide therapy.

Aortic root thrombi may result in systemic embolization. In the cited series, limb ischaemia was the most frequently reported presentation (84%), followed by visceral ischaemia (27%) and stroke (14%).^3^ Reported risk factors include assist devices, atherosclerosis, aneurysmal dilation, thrombophilia, malignancy, and interruption of anticoagulation. We describe a middle-aged man with prior venous thromboembolism who developed an aortic root thrombus complicated by cerebral and splenic infarcts, successfully managed with surgical excision.

## Summary figure

**Figure:**
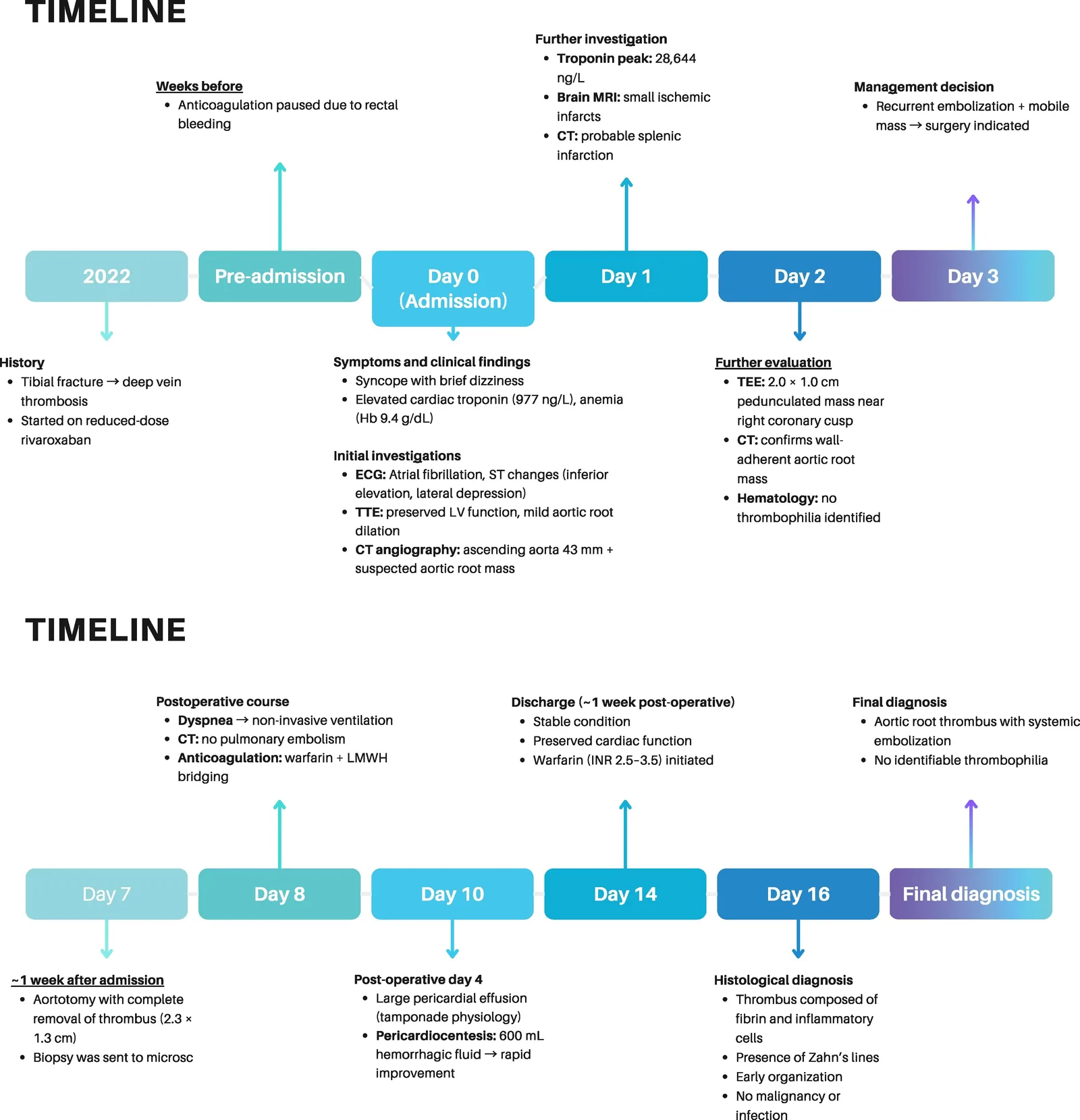


## Case presentation

A 47-year-old man was admitted in July 2025 after a syncopal episode, reporting brief dizziness without chest pain or prodromal symptoms. His history included deep vein thrombosis following a tibial fracture in 2022, treated with reduced-dose rivaroxaban (10 mg daily), recently paused due to rectal bleeding.

On admission, he was haemodynamically stable. Examination revealed irregular cardiac rhythm without murmurs, normal lung sounds, and no oedema.

Laboratory findings showed CRP 3 mg/L (ref: <6 mg/L), leukocytosis 12.0 × 10^9^/L (ref: 3.5–10.0 10^9^/L), thrombocytosis 446× 10^9^/L (ref: 145–348 × 10^9^/L), anaemia with Hb 9.4 g/dL (ref: 13.4–17.0 g/dL), elevated LD 331 U/L (ref: <205 U/L), NT-proBNP 96 ng/L (ref: <300 ng/L), D-dimer 0.5 (ref: <0.5 mg/L), and markedly elevated cardiac troponin I with 977 ng/L (ref: <3 ng/L) with otherwise unremarkable biochemistry.

ECG demonstrated new-onset atrial fibrillation (60 bpm), right bundle branch block-like pattern, ST depression in aVL and V2–V6, and inferior ST elevation, with spontaneous reversion to sinus rhythm.

Bedside echocardiography showed preserved left ventricular function and mild aortic root dilation without clear pathology. Notably, the aortic root mass was not visualized on transthoracic echocardiography despite its considerable size. CT angiography demonstrated dilation of the ascending aorta (43 mm) and a wall-adherent mass in the proximal aortic root near the right coronary ostium (*Figures 1 and 2*). This was an unusual location in the absence of dissection.

**Figure 1:**
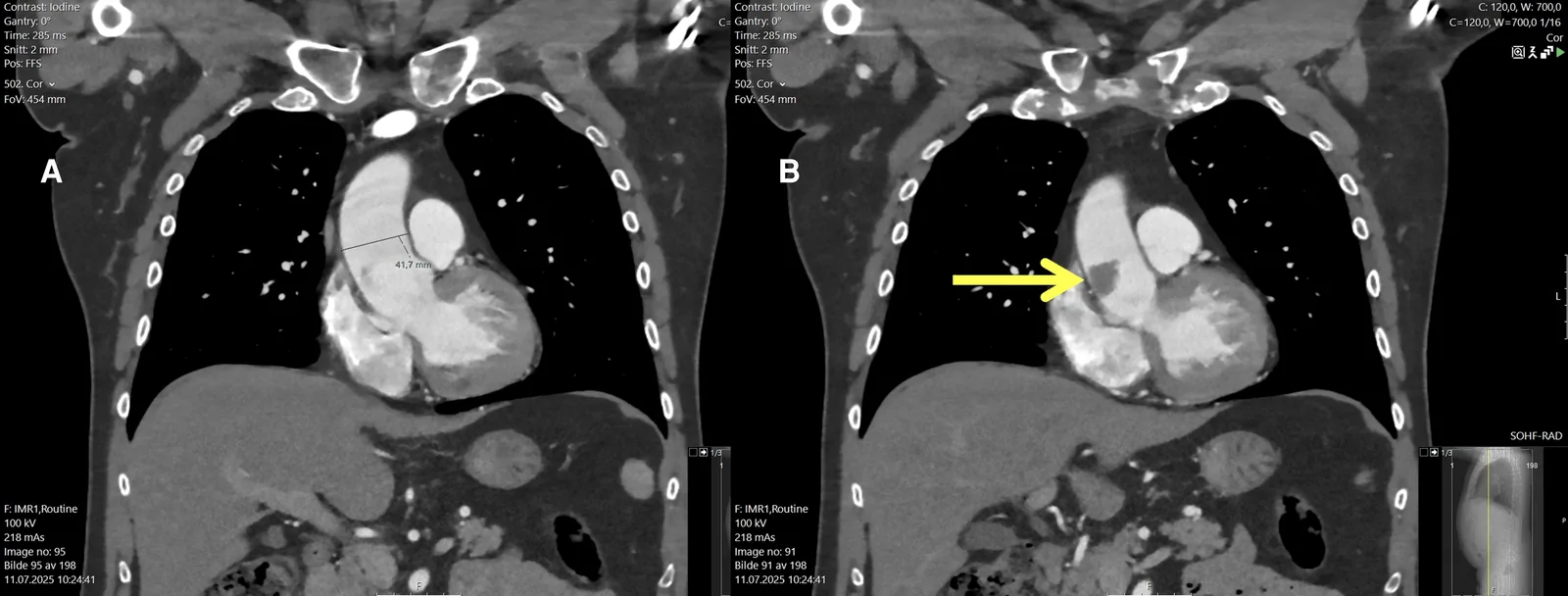
(*A*) CT sagittal plane demonstrating dilation of the ascending aorta measuring 41.7 mm. (*B*) CT image showing a mass (yellow arrow) in the ascending aorta.

**Figure 2:**
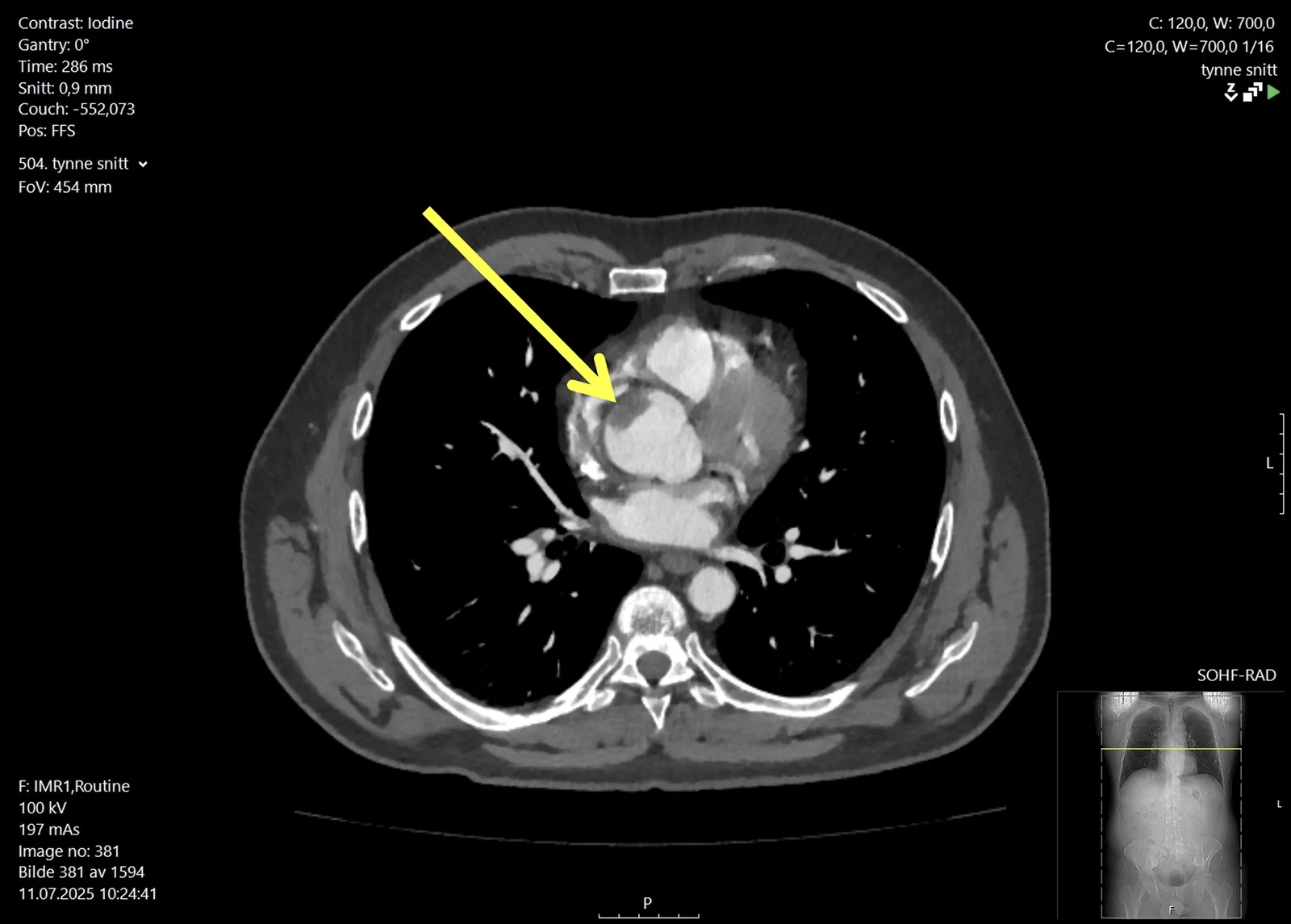
CT coronal plane showing the mass (yellow arrow) in the ascending aorta.

Troponin rose markedly (peak 28 644 ng/L), while haemoglobin dropped to 8.3 g/dL. Brain MRI showed two small ischaemic infarcts, and CT suggested splenic infarction.

Despite initial concern for acute coronary syndrome, preserved ventricular function and absence of regional wall motion abnormalities argued against myocardial infarction.

Anticoagulation with low molecular weight heparin (LMWH) was initiated cautiously due to anaemia, attributed to iron deficiency from haemorrhoidal bleeding.

Given recurrent embolic events, further evaluation was performed. Transoesophageal echocardiography demonstrated a 2.0 × 1.0 cm pedunculated mass attached to the sinus of the right coronary cusp. The lesion had a solid capsule with central hypoechogenicity, consistent with thrombus (*Figures 3 and 4*). The aortic valve was normal, and the root measured 47 mm.

**Figure 3:**
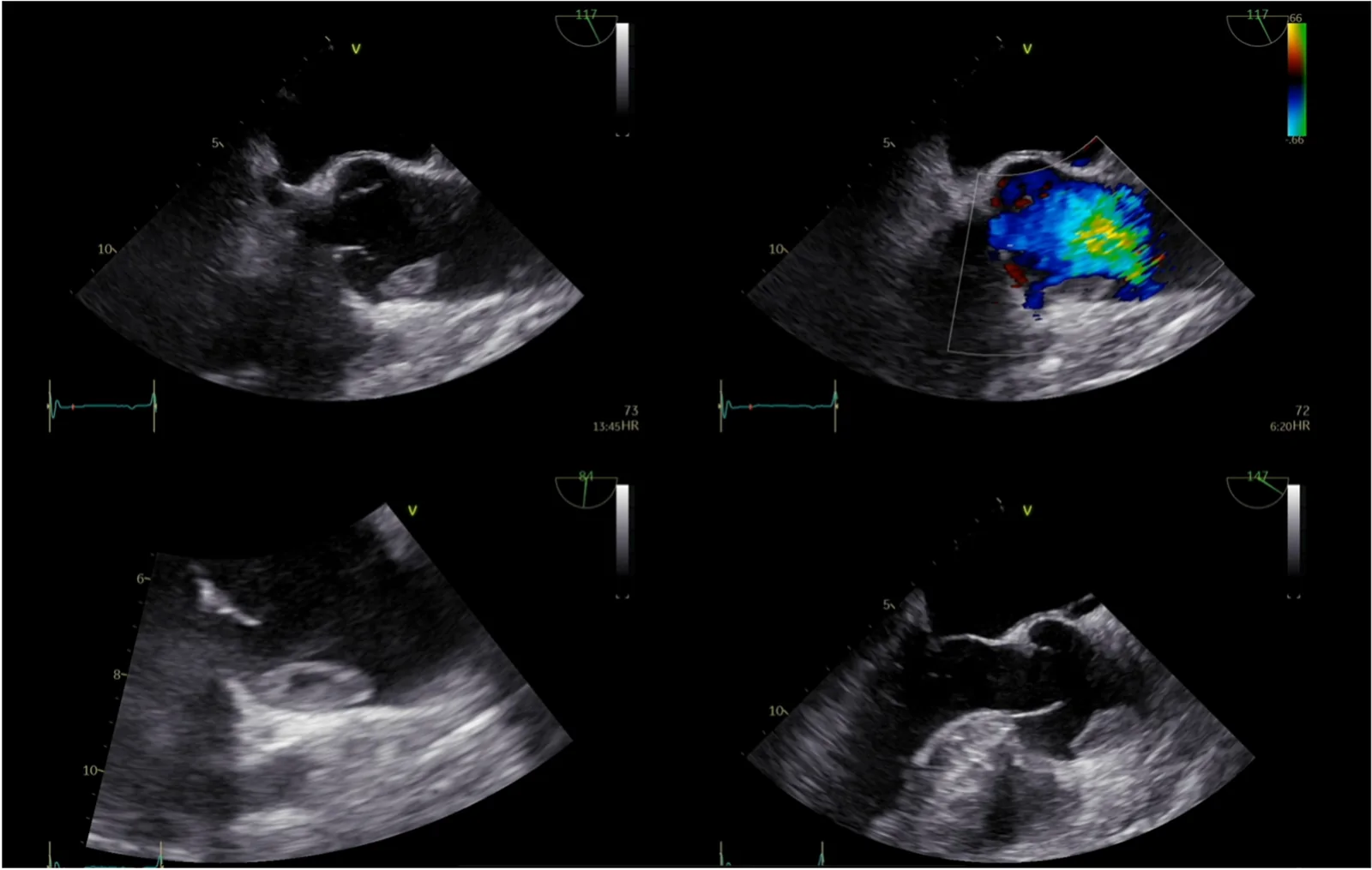
Transoesophageal echocardiography, short-axis view of the aorta (90–150 degrees), showing a dilated aortic root with a pedunculated hypoechogenic mural mass.

**Figure 4:**
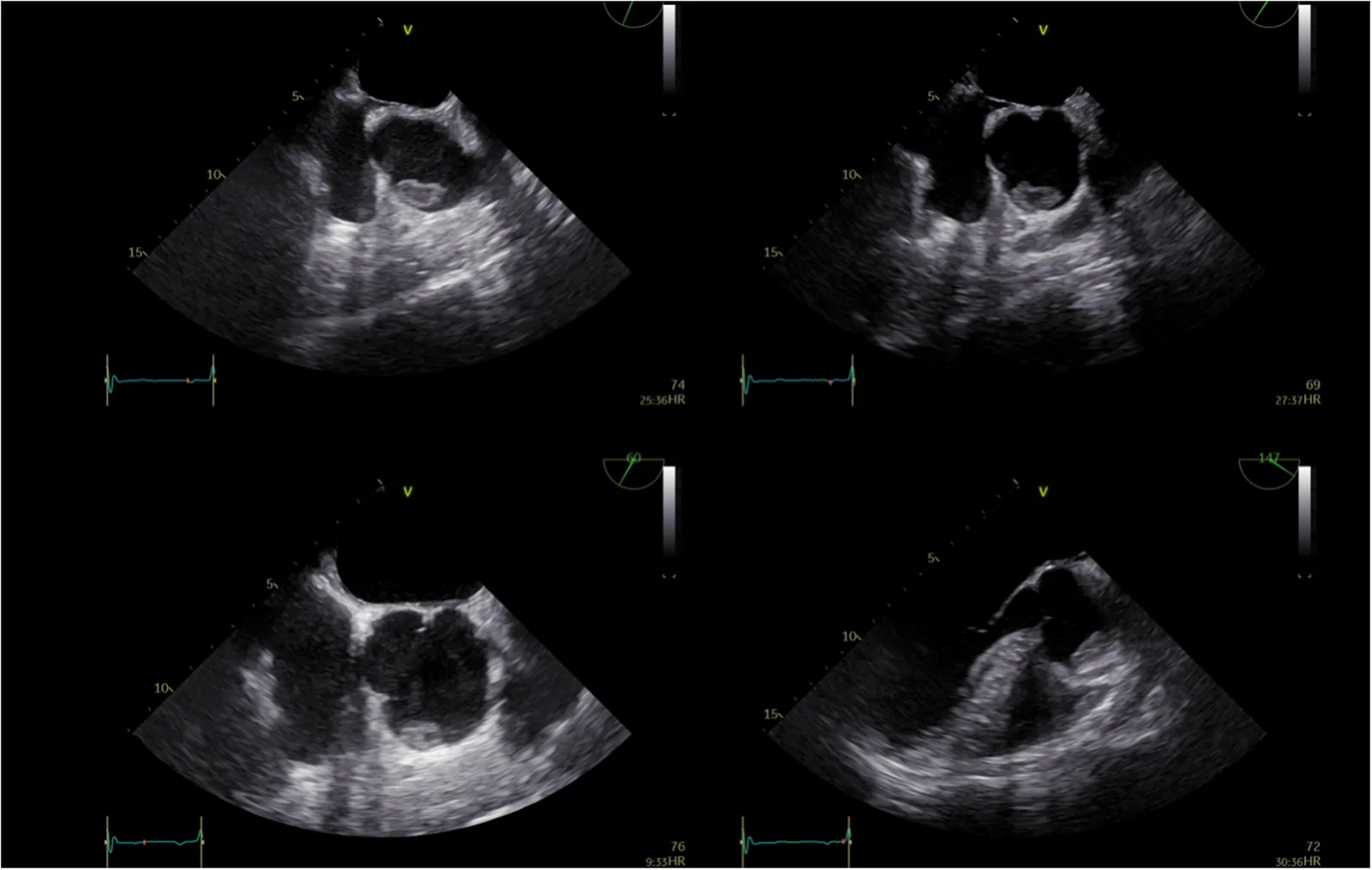
Transoesophageal echocardiography, short-axis view of the aorta (60–150 degrees), showing the mass attached to the sinus of the right coronary cusp.

The differential diagnoses included thrombus, papillary fibroelastoma, and myxoma. Haematologic work-up excluded antiphospholipid syndrome, myeloproliferative disease, and haemolysis; molecular testing (JAK2, CALR, MPL, BCR-ABL) was negative. Given recurrent embolization and a large mobile mass, surgery was recommended.

One week later, aortotomy was performed with complete removal of a 2.3 × 1.3 cm pedunculated mass above the right coronary cusp. The mass was easily dissected, and the aortic wall showed only focal early calcific changes, without ulceration at the site of thrombus attachment.

Postoperatively, he developed dyspnoea requiring non-invasive ventilation; CT excluded pulmonary embolism. Warfarin was initiated with LMWH bridging. Four days later, echocardiography revealed a large pericardial effusion (27 mm) with tamponade physiology, requiring drainage of 600 mL of haemorrhagic fluid with rapid resolution.

At discharge 1 week later, biventricular function was preserved with minimal residual effusion. Haemoglobin stabilized at 11 g/dL. He was discharged on warfarin (INR 2.5–3.5) with planned follow-up imaging.

Microbiology was negative. Histology confirmed thrombus with fibrin, inflammatory cells, Zahn’s lines, and early organization, without malignancy.

## Discussion

Aortic root thrombus is rare and may mimic acute coronary syndrome or inflammatory disease. In this case, embolic infarcts, elevated troponin, and transient ECG changes initially suggested myocardial infarction. However, preserved ventricular function and low inflammatory markers argued against this.

The pathogenesis of the aortic root thrombus in this patient was likely multifactorial. No underlying thrombophilia or myeloproliferative disorder was identified. However, mild thrombocytosis may have contributed to a prothrombotic state, while interruption of anticoagulation may have further increased the risk of thrombus formation. In addition, aortic root dilation may have altered local blood flow and promoted thrombus formation. Although focal early calcific changes of the aortic wall were observed intraoperatively, there was no ulceration at the site of thrombus attachment. Taken together, these factors may have contributed to thrombus formation despite the absence of an identifiable systemic thrombophilic disorder.

Given recurrent embolization and high-risk morphology, surgical removal was favoured over anticoagulation alone, particularly due to bleeding risk. Surgery was curative but complicated by pericardial effusion, highlighting the need for close postoperative monitoring.

Primary cardiac tumours must be considered in the differential. Most are benign, with myxomas accounting for ∼50%. Papillary fibroelastomas are the most common valvular tumours and may embolize, while malignant tumours are rare but aggressive.^5,6^

Although uncommon, papillary fibroelastomas arising from the aortic wall have been reported, as have other aortic wall masses including thrombi and sarcomas.^4,7–9^ Vascular myxomas are exceedingly rare but described.^10^ Surgical removal is typically required due to embolic risk. Cardiac MRI has high diagnostic accuracy for mass characterization.^11^ However, ongoing embolization in this case necessitated immediate surgical intervention.

This case highlights that aortic root thrombus should be considered in patients with systemic embolization, particularly in the setting of anticoagulation interruption and aortic dilation.

## Conclusion

Aortic root thrombus is a rare but potentially life-threatening condition that may present with syncope, myocardial injury, and systemic embolization, requiring high clinical suspicion and multimodal imaging for diagnosis.

Large, mobile thrombi with high embolic risk require prompt surgical removal. Multidisciplinary management, close postoperative monitoring, and long-term anticoagulation with imaging follow-up are essential to reduce recurrence and optimize outcomes. Identifying the underlying cause remains key to guiding prophylactic therapy.

## Lead author biography


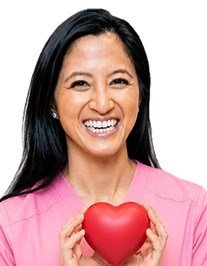
Ai Phi Thuy Ho, MD, is a physician in cardiology with a particular interest in cardiovascular imaging and echocardiography. Alongside her clinical work, she is actively engaged in medical innovation, research, and education, with a focus on advancing early diagnosis and preventive care. Her professional interests include cardiovascular disease, women’s health, and emerging medical technologies aimed at improving patient care and our understanding of human health.

**Consent:** Written informed consent was obtained from the patient for publication of this case report and accompanying images in accordance with COPE guidelines.

## Data Availability

The CT and echocardiographic data used in this case report are stored in the electronic patient records by a third party. Access to these data may be available upon reasonable request after permission has been obtained from both the data holder and the patient.
